# Supplementary figures and images for: β-1,6-Glucan plays a central role in the structure and remodeling of the bilaminate fungal cell wall
Source: eLife. 2024 Dec 5;13:RP100569. doi: 10.7554/eLife.100569 (PMC11620752; doi:10.7554/eLife.100569)

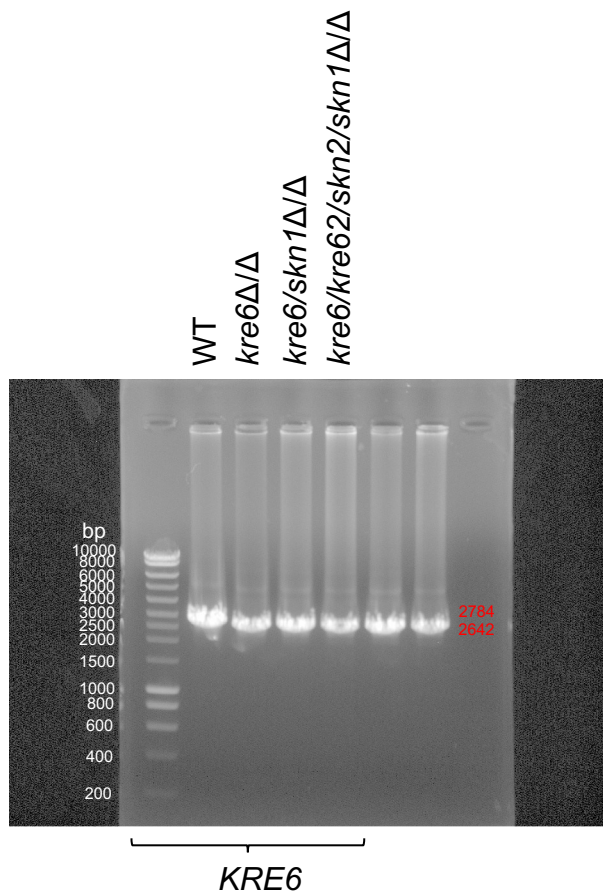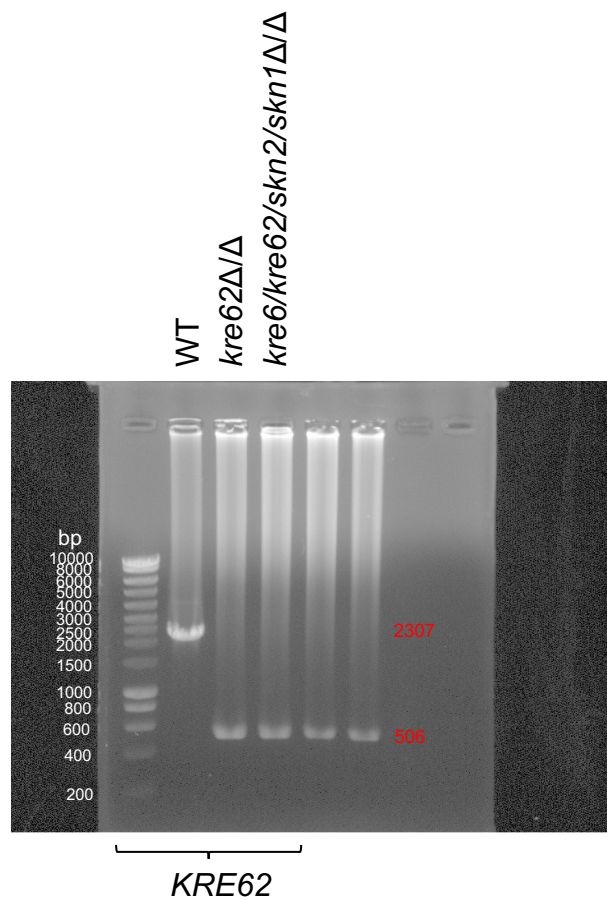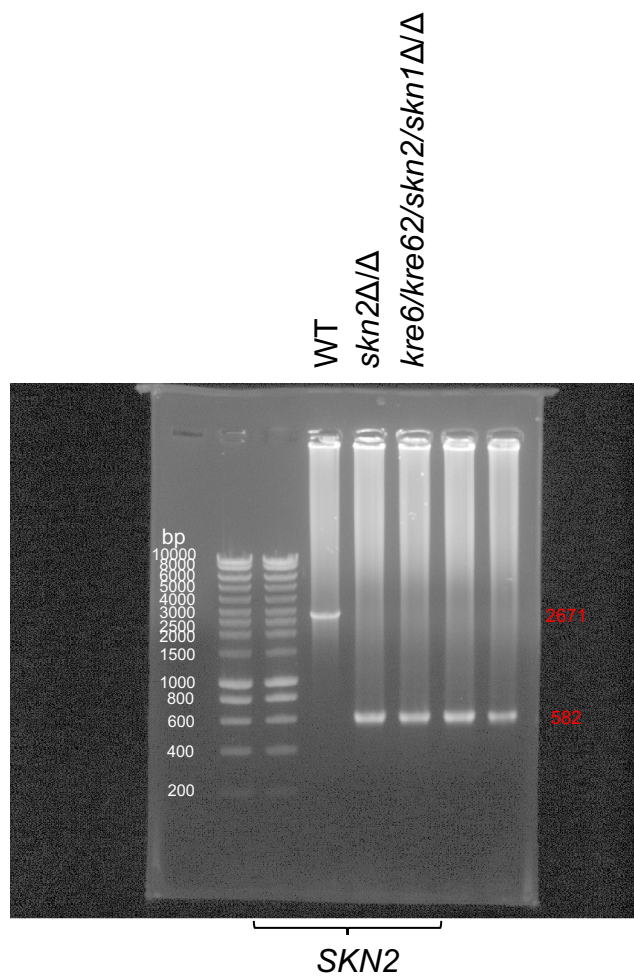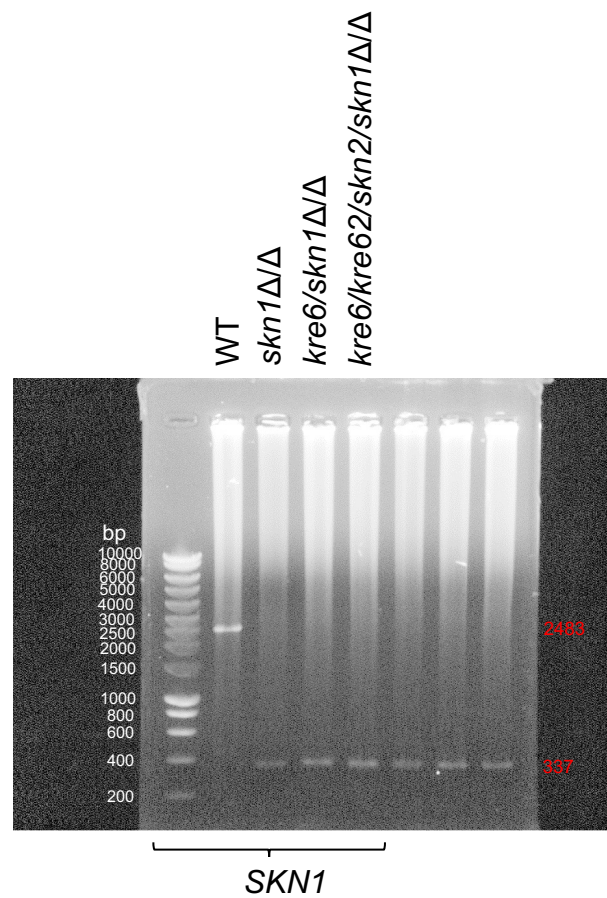

Supplement: Figure 4—figure supplement 1—source data 1. [file elife-100569-fig4-figsupp1-data1.zip › Figure 4-figure supplement 1-Source Data 1/Panel c.pdf]

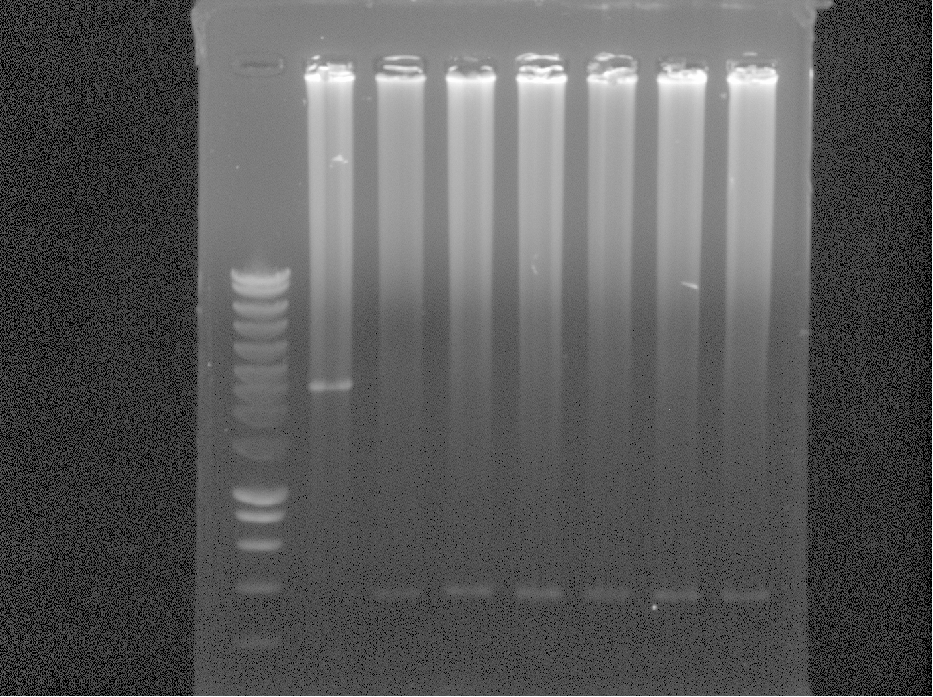

Supplement: Figure 4—figure supplement 1—source data 2. [file elife-100569-fig4-figsupp1-data2.zip › Figure 4-figure supplement 1-Source Data 2/Panel c SKN1.tif]

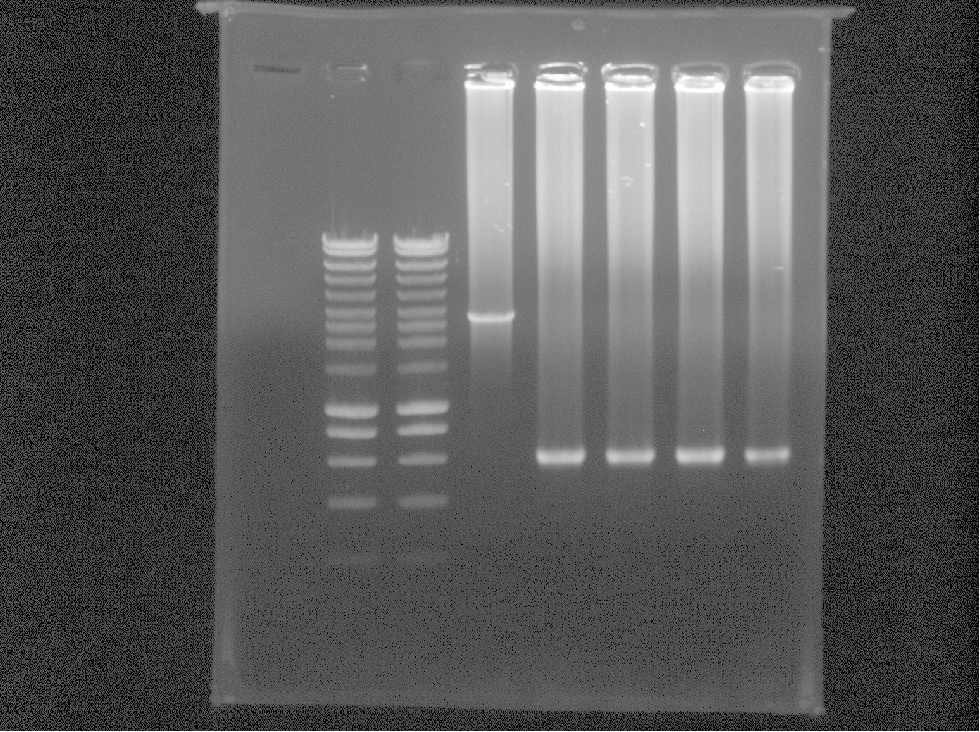

Supplement: Figure 4—figure supplement 1—source data 2. [file elife-100569-fig4-figsupp1-data2.zip › Figure 4-figure supplement 1-Source Data 2/Panel c SKN2.tif]

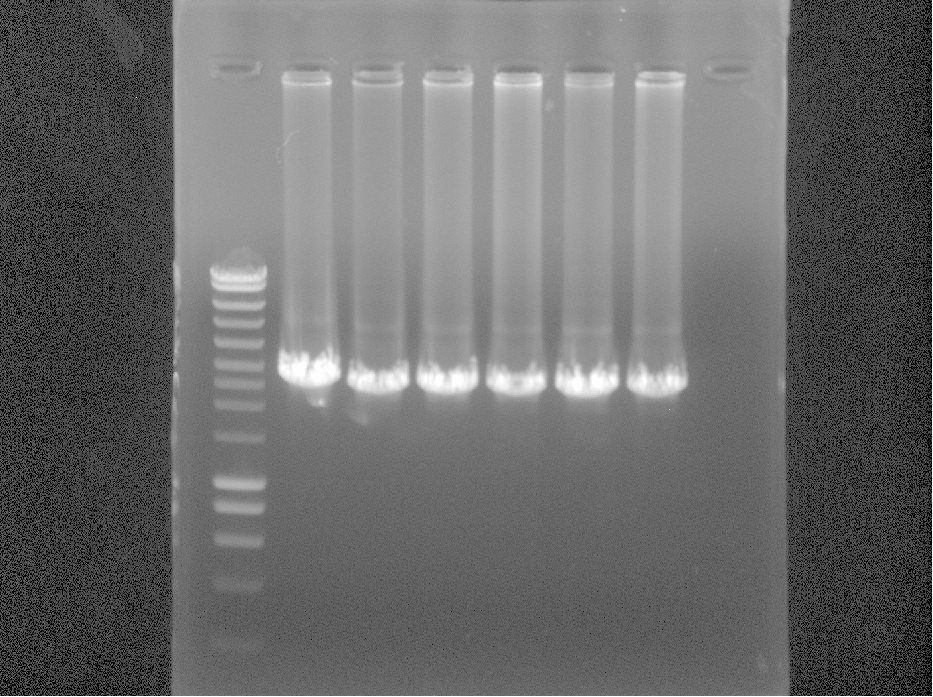

Supplement: Figure 4—figure supplement 1—source data 2. [file elife-100569-fig4-figsupp1-data2.zip › Figure 4-figure supplement 1-Source Data 2/Panel c KRE6.tif]

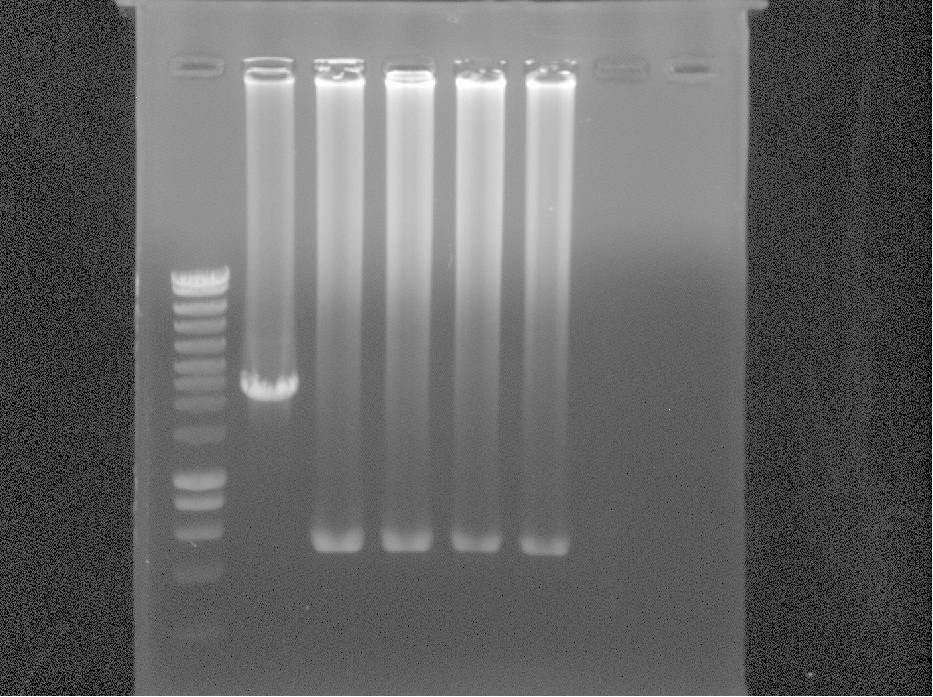

Supplement: Figure 4—figure supplement 1—source data 2. [file elife-100569-fig4-figsupp1-data2.zip › Figure 4-figure supplement 1-Source Data 2/Panel c KRE62.tif]

*kre6/kre62/skn2/skn1Δ/Δ + P<sub>ACT1</sub>-KRE6*

WT

bp

10000  
8000  
6000  
5000  
4000  
3000  
2500  
2000  
1500  
1000  
800  
600  
400  
200

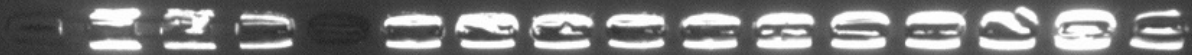

Supplement: Figure 4—figure supplement 2—source data 1. [file elife-100569-fig4-figsupp2-data1.zip › Figure 4-figure supplement 2-Source Data 1/Control PCR quadruple mutant complemented KRE6.pdf]

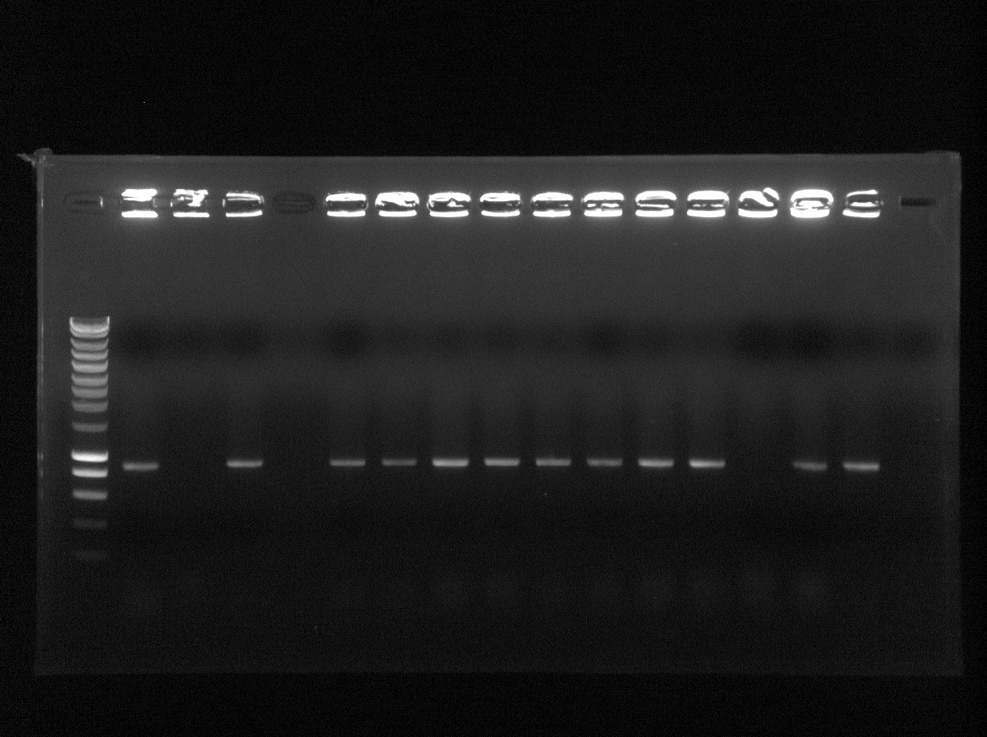

Supplement: Figure 4—figure supplement 2—source data 2. [file elife-100569-fig4-figsupp2-data2.zip › Figure 4-figure supplement 2-Source Data 2/Control PCR quadruple mutant complemented KRE6.tiff]

Medium

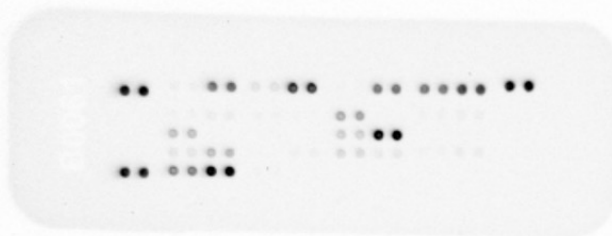

AI Fraction

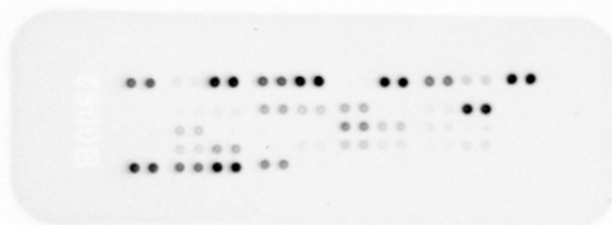

AI-OxP Fraction

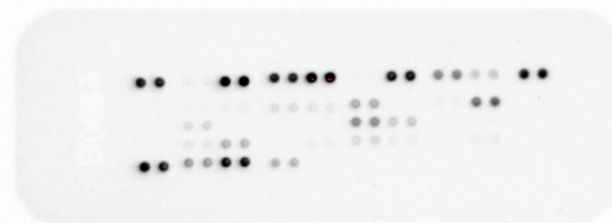

$\beta$ -1,6-glucan

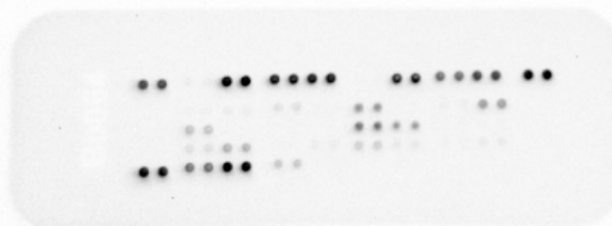

Supplement: Figure 6—figure supplement 2—source data 1. [file elife-100569-fig6-figsupp2-data1.zip › Figure 6-figure supplement 2-Source Data 1/Human proteome profiler PBMCs.pdf]

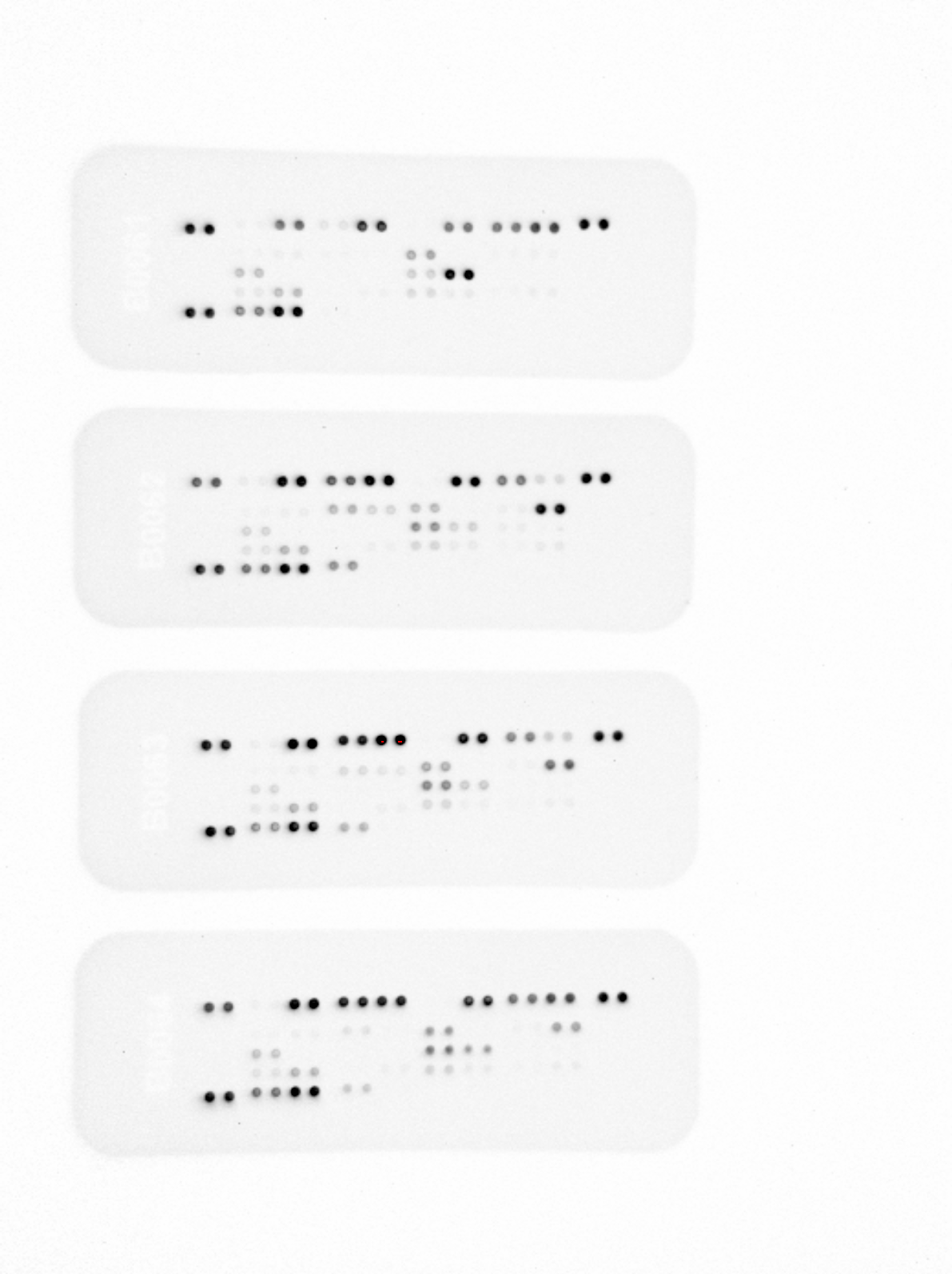

Supplement: Figure 6—figure supplement 2—source data 2. [file elife-100569-fig6-figsupp2-data2.zip › Figure 6-figure supplement 2-Source Data 2/Human proteome profiler PBMCs.tif]

Medium

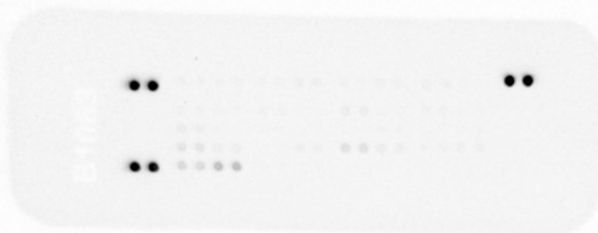

AI Fraction

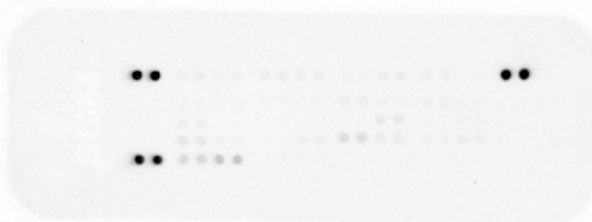

AI-OxP Fraction

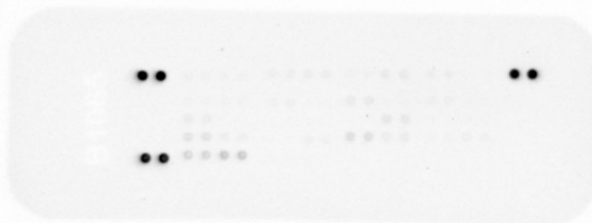

$\beta$ -1,6-glucan

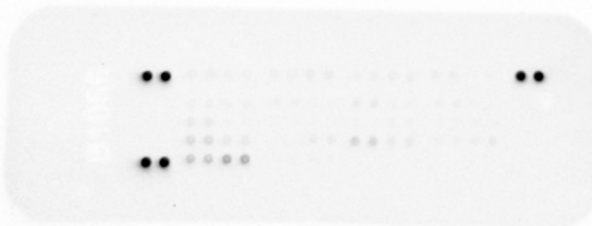

Supplement: Figure 6—figure supplement 3—source data 1. [file elife-100569-fig6-figsupp3-data1.zip › Figure 6-figure supplement 3-Source Data 1/Human proteome profiler neutrophils.pdf]

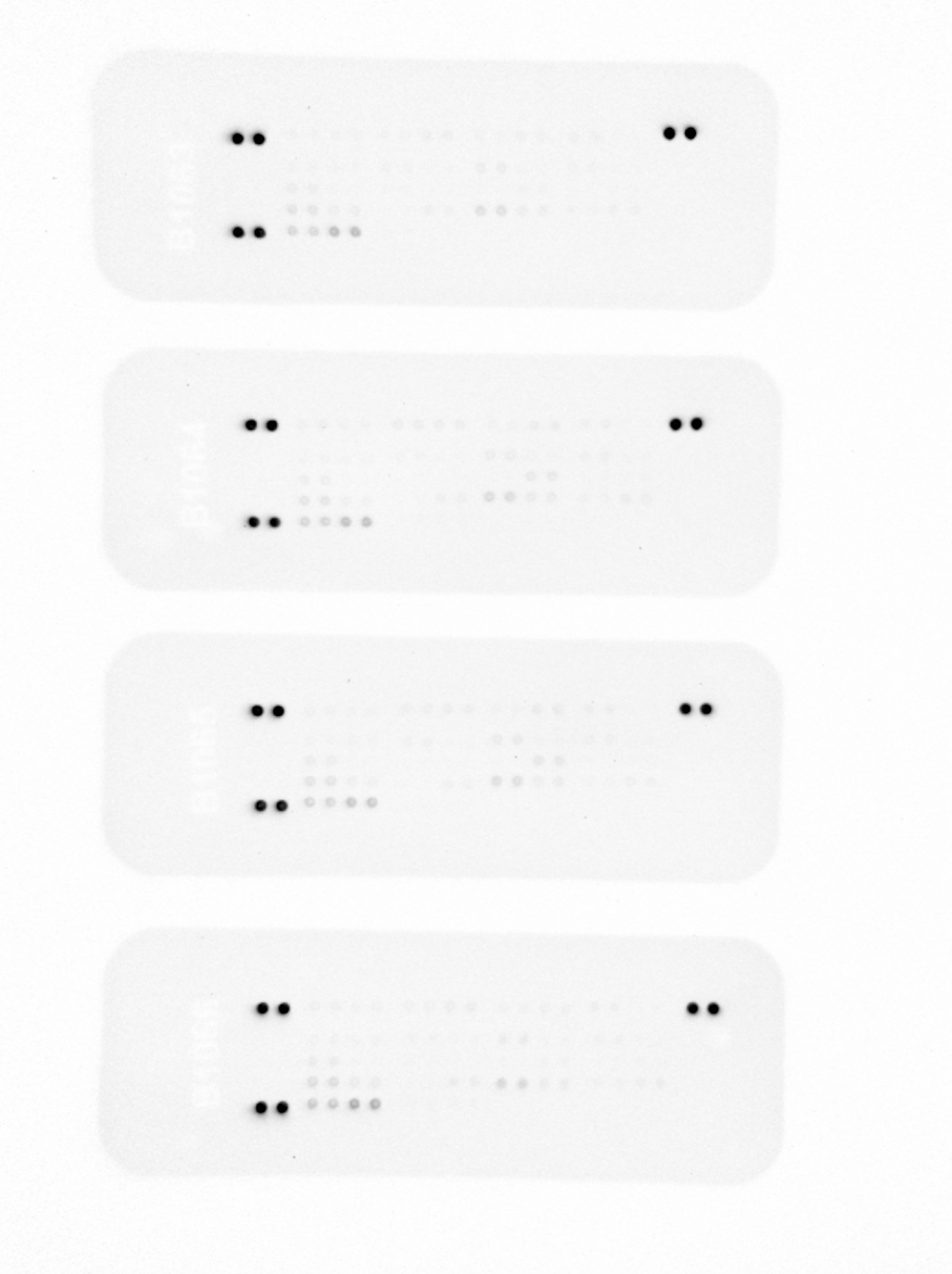

Supplement: Figure 6—figure supplement 3—source data 2. [file elife-100569-fig6-figsupp3-data2.zip › Figure 6-figure supplement 3-Source Data 2/Human proteome profiler neutrophils.tif]
