# Supplementary material for: β-1,6-Glucan plays a central role in the structure and remodeling of the bilaminate fungal cell wall
Source: eLife. 2024 Dec 5;13:RP100569. doi: 10.7554/eLife.100569 (PMC11620752; doi:10.7554/eLife.100569)
Supplement: Supplementary file 1. [file elife-100569-supp1.docx]

**Supplementary File 1: *C. albicans* strains used in this study.**

| **Strain name** | **Parental strain** | **Relevant genotype** | **Reference** |
| --- | --- | --- | --- |
| SC5314 | - | Clinical blood isolate | PMID: 6394964 |
| CAF2-1 | SC5314 | *URA3/ura3*Δ*::imm434* | PMID: 8349105 |
| CAI4 | CAF2-1 | *ura3*Δ*::imm434/ura3*Δ*::imm434* | PMID: 8349105 |
| BWP17 | CAI4 | *ura3*Δ*::imm434/ura3*Δ*::imm434 his1::hisG/his1::hisG arg4::hisG/arg4::hisG* | PMID: 10074081 |
| SN152 | CAI4 | *arg4*Δ*/arg4*Δ *leu2*Δ*/leu2*Δ *his1*Δ*/his1*Δ *URA3/ura3*Δ*::imm434 IRO1/iro1*Δ*::imm434* | PMID: 15701792 |
| *cwh41*Δ/Δ | CAI4 | Same as CAI4 but *cwh41*Δ*::dp1200/cwh41*Δ*::dp1200 RPS1/rps1*Δ*::*CIp10 | PMID: 17933909 |
| *rot2*Δ/Δ | CAI4 | Same as CAI4 but *rot2*Δ*::dp1200/rot2*Δ*::dp1200 RPS1/rps1*Δ*::*CIp10 | PMID: 17933909 |
| *kre5*Δ/Δ | SN152 | Same as SN152 but *kre5*Δ*::leu2/kre5*Δ*::his1* | PMID: 20543849 |
| *kre6*Δ/Δ | SC5314 | *kre6*Δ*::HygB/kre6*Δ*::HygB* | This study |
| *kre62*Δ/Δ | SC5314 | *kre62*Δ*::FRT/kre62*Δ*::FRT* | This study |
| *skn2*Δ/Δ | SC5314 | *skn2*Δ*::FRT/skn2*Δ*::FRT* | This study |
| *skn1*Δ/Δ | SC5314 | *skn1*Δ*::FRT/skn1*Δ*::FRT* | This study |
| *kre6/skn1*Δ/Δ | *kre6Δ/Δ* | *kre6*Δ*::HygB/kre6*Δ*::HygB skn1*Δ*::FRT/skn1*Δ*::FRT* | This study |
| *kre6/kre62/skn2/skn1*Δ/Δ | *kre6Δ/Δ* | *kre6*Δ*::HygB/kre6*Δ*::HygB kre62*Δ*::FRT/kre62*Δ*::FRT skn2*Δ*::FRT/skn2*Δ*::FRT skn1*Δ*::FRT/skn1*Δ*::FRT* | This study |
| *kre6/kre62/skn2/skn1*Δ/Δ*+*P*_ACT1_-KRE6* | *kre6/kre62/skn2/skn1Δ/Δ* | *kre6*Δ*::HygB/kre6*Δ*::HygB kre62*Δ*::FRT/kre62*Δ*::FRT skn2*Δ*::FRT/skn2*Δ*::FRT skn1*Δ*::FRT/skn1*Δ*::FRT RPS1/RPS1::*CIp*SAT1-*P*_ACT1_-KRE6* | This study |
| *kre1*Δ/Δ | BWP17 | Same as BWP17 but *kre1*Δ*::arg4/kre1*Δ*::his1* | Provided by Mathias Richard |
| P*_MRP1_-CHS1/chs1*Δ | CAI4 | Same as CAI4 but *chs1*Δ*::hisG/chs1*Δ*:*pSK*-URA3-*P*_MRP1_-CHS1* | PMID: 11251855 |
| *chs2*Δ/Δ | CAF2-1 | Same as CAF2-1 but *chs2*Δ*::hisG/chs2*Δ*::hisG-URA3-hisG* | PMID: 8636047 |
| *chs3*Δ/Δ | CAF2-1 | Same as CAF2-1 but *chs3-2::hisG/chs3-3::hisG-URA3-hisG* | PMID: 7479842 |
| *mnt1/mnt2*Δ/Δ | CAI4 | Same as CAF2-1 but *mnt1-mnt2*Δ*::hisG/mnt1-mnt2*Δ*::hisG-URA3-hisG* | PMID: 15519997 |
| *mnn2/22/21/23/24/26*Δ/Δ | CAI4 | Same as CAF2-1 but *mnn2*Δ*::dpl200/mnn2*Δ*::dpl200 mnn22*Δ*::dpl200/mnn2*Δ*::dpl200 mnn23*Δ*::dpl200/mnn23*Δ*::dpl200 mnn24*Δ*::dpl200/mnn24*Δ*::dpl200 mnn26*Δ*::dpl200/mnn26*Δ*::dpl200 mnn21*Δ*::dpl200/mnn21*Δ*::dpl200* | PMID: 23633946 |
| *mnn9*Δ/Δ | CAI4 | Same *as CAF2-1 mnn9*Δ*::hisG/mnn9*Δ*::hisG* Δ*ura3*Δ*::imm434/ura3*Δ*::imm434* | PMID: 10601199 |
| *och1*Δ/Δ | SN152 | Same as SN152 but *och1*Δ*::leu2/och1*Δ*::his1* | PMID: 20543849 |
| *fks1*Δ | SC5314 | SC5314, but *fks1/fks1*Δ | [PMID: 30370375](https://pubmed.ncbi.nlm.nih.gov/30370375) |
| *phr1*Δ/Δ | BWP17 | Same as BWP17 but *phr1*Δ*::hisG/phr1*Δ | PMID: 7823929 |
| *phr2*Δ/Δ | BWP17 | Same as BWP17 but *phr2*Δ*::hisG/phr2*Δ*::hisG-URA3-hisG* | PMID: 9315654 |
