# Supplementary material for: β-1,6-Glucan plays a central role in the structure and remodeling of the bilaminate fungal cell wall
Source: eLife. 2024 Dec 5;13:RP100569. doi: 10.7554/eLife.100569 (PMC11620752; doi:10.7554/eLife.100569)
Supplement: Supplementary file 2. [file elife-100569-supp2.docx]

**Supplementary File 2: Primers used in this study.**

| **Primer name** | **Sequence (5' -> 3')** | **Remarks** |
| --- | --- | --- |
| SNR52/F | AAGAAAGAAAGAAAACCAGGAGTGAA | Forward primer for the amplification of SNR52 promoter (Min 2016) |
| sgRNA/R | ACAAATATTTAAACTCGGGACCTGG | Reverse primer for the amplification of sgRNA scaffold (Min 2016) |
| SNR52/N | GCGGCCGCAAGTGATTAGACT | Forward and reverse primers for nested PCR (for construction of sgRNA expression cassette; Min 2016) |
| sgRNA/N | GCAGCTCAGTGATTAAGAGTAAAGATGG |  |
| CaCas9/F | ATCTCATTAGATTTGGAACTTGTGGGTT | Forward primer for amplifying the CaCas9 cassette (Min 2016) |
| CaCas9/R | TTCGAGCGTCCCAAAACCTTCT | Reverse primer for amplifying the CaCas9 cassette (Min 2016) |
| SNR52/R/SKN1 | CGAGTCATTTCGTCTTGGTTCAAATTAAAAATAGTTTACGCAAGTC | Reverse primers for the amplification of SNR52 promoter with overlapping guide sequence of the target gene.  Guide sequence in red. |
| SNR52/R/SKN2 | TTCGATTCTAGCAACAGACTCAAATTAAAAATAGTTTACGCAAGTC |  |
| SNR52/R/KRE62 | GATATATTACTTGAATCACTCAAATTAAAAATAGTTTACGCAAGTC |  |
| SNR52/R/KRE6 | CTATAAGCTTGGAATGGTTTCAAATTAAAAATAGTTTACGCAAGTC |  |
| sgRNA/F/SKN1 | AACCAAGACGAAATGACTCGGTTTTAGAGCTAGAAATAGCAAGTTAAA | Forward primers for the amplification of sgRNA scaffold with overlapping guide sequence of the target gene.  Guide sequence in red. |
| sgRNA/F/SKN2 | AGTCTGTTGCTAGAATCGAAGTTTTAGAGCTAGAAATAGCAAGTTAAA |  |
| sgRNA/F/KRE62 | AGTGATTCAAGTAATATATCGTTTTAGAGCTAGAAATAGCAAGTTAAA |  |
| sgRNA/F/KRE6 | AAACCATTCCAAGCTTATAGGTTTTAGAGCTAGAAATAGCAAGTTAAA |  |
| SAT1FLP/F/SKN1 | ACTACCACTACTATTATAGAAATTTTTATCATATATATACAATTGGCTACTAAAAACTTAAA  CTTTTAATACAACTTACAGTACCGGGCCCCCCCTCGA | Primers to amplify the repair template (*SAT1*-Flipper or HygR) |
| SAT1FLP/F/SKN2 | ATAATAGATTTTGTTTTTAAACGGATAACCCTTCCATTCCATTCTAGCAAACCAAAAGTAAA  TCAAAAGAACTAACTACCGTACCGGGCCCCCCCTCGA |  |
| SAT1FLP/F/KRE62 | AATATTCAACAACTATTCTTTCTACTTCTTTTAAAGAACCCGAACTTTTTTTTTGACTTTATAA  TAATTAATGTATCATTGTACCGGGCCCCCCCTCGA |  |
| HygR/F/KRE6 | GATAAGACCTCAAAATGGCGTCTCAAAGAGAGATGGAATGAATGGGATGAATCATCAAACAAGAG |  |
| SAT1FLP/R/SKN1 | AATTATATACAATGAATGAATGAATGAAAATGTCTATTTAAAAGTATATAAAAATATG  TAAATATAGGGGGGTTGGTGTTGGCCGCTCTAGAACTAGTGGAT |  |
| SAT1FLP/R/SKN2 | CAGAGAACACATGAAGGGTGGTAAACTATTTTGTCAGTTTTTTTTCTCCTTTTTTTTAGT  ACGTGGAGTTCGCCTTTAATGGCCGCTCTAGAACTAGTGGAT |  |
| SAT1FLP/R/KRE62 | ATCGTCTTGTTTGAGATTGTTCTTTTAACTTTTGTTCTACACTACCACCACCACCACCATC  ATTGTTATTCTTGTTGTTGGGCCGCTCTAGAACTAGTGGAT |  |
| hygR/R/KRE6 | AGTAGTAGTAGTAATAGTAGTACCATCGCCACCTGACGTCGTATAGTGCTTGCTGTTCG |  |
| Flanking_F/SKN1 | CTACAGTTTATAGAAGTTAATACCAGTACC |  |
| Flanking_F/SKN2 | ATATCCCGCTCCATCCATTC |  |
| Flanking_F/KRE62 | AGGCTAGGCTAAGTATTAAC |  |
| Flanking_R/SKN1 | CATTATCATCAGGTCAATTTGACTCAT |  |
| Flanking_R/SKN2 | ACCACTGTCGTACAAAATTGC |  |
| Flanking_R/KRE62 | ATTACCTGATTTGGTGATGC |  |
| screenKre6/F | ACTTGGCAACATTTGCAACA |  |
| screenKre6/R | TCTGGGGTCACTTTCTTAAAATCCT |  |
| CIpUL | ATACTACTGAAAATTTCCTGACTTTC |  |
| CIpSAT | CTAGTGGATCCCCCAGATCATTATCC |  |
| KRE6-FWD | GGGGACAAGTTTGTACAAAAAAGCAGGCTtgATGGCGTCTCAAAGAGATTTAACTTCAAAT | Primers used for cloning KRE6 in pDONR207 |
| KRE6-REV | GGGGACCACTTTGTACAAGAAAGCTGGGTcTTAACATCCAATTAAACTATTTTTAGGGAA |  |
